# Possible Role of Envelope Components in the Extreme Copper Resistance of the Biomining *Acidithiobacillus ferrooxidans*

**DOI:** 10.3390/genes9070347

**Published:** 2018-07-10

**Authors:** Nia Oetiker, Rodrigo Norambuena, Cristóbal Martínez-Bussenius, Claudio A. Navarro, Fernando Amaya, Sergio A. Álvarez, Alberto Paradela, Carlos A. Jerez

**Affiliations:** 1 Laboratory of Molecular Microbiology and Biotechnology, Department of Biology, Faculty of Sciences, University of Chile, Santiago 7800003, Chile; nia.oetiker.mancilla@gmail.com (N.O.); rodrigoanv93@gmail.com (R.N.); cm.bussenius@gmail.com (C.M.-B.); clnavarrol@gmail.com (C.A.N.); 2 Department of Biochemistry and Molecular Biology, Faculty of Chemical and Pharmaceutical Sciences, University of Chile, Santiago 7800003, Chile; fernando.amaya@ug.uchile.cl (F.A.); salvarez@uchile.com (S.A.Á.); 3 Proteomics Laboratory, National Biotechnology Center, CSIC, 28049 Madrid, Spain; alberto.paradela@cnb.csic.es

**Keywords:** *Acidithiobacillus ferrooxidans*, copper resistance, biomining, envelope components, proteomics, lipopolysaccharide

## Abstract

*Acidithiobacillus ferrooxidans* resists extremely high concentrations of copper. Strain ATCC 53993 is much more resistant to the metal compared with strain ATCC 23270, possibly due to the presence of a genomic island in the former one. The global response of strain ATCC 53993 to copper was analyzed using iTRAQ (isobaric tag for relative and absolute quantitation) quantitative proteomics. Sixty-seven proteins changed their levels of synthesis in the presence of the metal. On addition of CusCBA efflux system proteins, increased levels of other envelope proteins, such as a putative periplasmic glucan biosynthesis protein (MdoG) involved in the osmoregulated synthesis of glucans and a putative antigen O polymerase (Wzy), were seen in the presence of copper. The expression of *A. ferrooxidans* *mdoG* or *wzy* genes in a copper sensitive *Escherichia coli* conferred it a higher metal resistance, suggesting the possible role of these components in copper resistance of *A. ferrooxidans*. Transcriptional levels of genes *wzy*, *rfaE* and *wzz* also increased in strain ATCC 23270 grown in the presence of copper, but not in strain ATCC 53993. Additionally, in the absence of this metal, lipopolysaccharide (LPS) amounts were 3-fold higher in *A. ferrooxidans* ATCC 53993 compared with strain 23270. Nevertheless, both strains grown in the presence of copper contained similar LPS quantities, suggesting that strain 23270 synthesizes higher amounts of LPS to resist the metal. On the other hand, several porins diminished their levels in the presence of copper. The data presented here point to an essential role for several envelope components in the extreme copper resistance by this industrially important acidophilic bacterium.

## 1. Introduction

*Acidithiobacillus ferrooxidans* is a gram-negative, acidophilic, chemolithoautotrophic bacterium able to use ferrous iron, reduced species of sulfur or metal sulfides as energy sources [1,2,3,4,5]. These bacteria are able to grow at high concentrations of several metals. This is an important property since they are used in biomining processes where copper concentrations are in the range of 15 to 100 mM [6,7,8]. Furthermore, these microorganisms can be used to exploit these natural resources sustainably [9]. Current knowledge indicates that *A. ferrooxidans* uses key elements involved in copper resistance in all bacteria [10,11,12], but in addition, it may have a broader repertoire of these known copper resistance determinants [13,14].

In the biomining environment, copper and other toxic metals are present in concentrations that are one to two orders of magnitude greater than those tolerated by neutrophils [6,15,16,17,18]. Most likely, the microorganisms forming part of the biomining consortium have developed additional strategies to resist the harsh conditions in which they live, and their study is therefore of great interest [19,20].

*A. ferrooxidans* ATCC 53993 is much more resistant to copper and other metals than *A. ferrooxidans* ATCC 23270. Both strains have the same copper resistance determinants but strain ATCC 53993 contains a genomic island (GI) having 160 extra genes, some of which code for additional copies of proteins involved in copper tolerance [13,14].

The response to copper of both *A. ferrooxidans* ATCC 53993 and ATCC 23270 was previously compared at 40 mM CuSO_4_ [14,21,22]. These preliminary studies were done by using ICPL (isotope-coded protein labeling) quantitative proteomics [22] and showed that strain ATCC 23270 synthesized much more oxidative-stress-related proteins than strain 53993 in response to copper, clearly indicating that the former strain is much more sensitive to the metal [22]. A high overexpression of RND (Resistance-Nodulation-Division) efflux systems and copper periplasmic chaperones CusF were seen in both strains subjected to copper. However, in strain ATCC 53993 both of its additional genes present in its genomic island were also overexpressed. This behavior suggested a possible explanation for the much higher copper resistance of strain ATCC 53993. In addition, changes in the levels of the respiratory system copper-binding proteins AcoP, Rus and several other proteins with predicted functions suggested that numerous metabolic changes are involved in controlling the effects of the toxic metal in strain ATCC 53993 [22].

To understand in more detail the reason by which *A. ferrooxidans* ATCC 53993 stands higher copper concentrations compared with strain 23270, iTRAQ (isobaric tag for relative and absolute quantitation) proteomics, transcriptional expression of genes of interest and functional assays were used in the current report. Increased levels of novel possible copper resistance determinants present in the envelope of *A. ferrooxidans* ATCC 53993 such as outer membrane proteins, the periplasmic glucans synthesizing protein MdoG and proteins involved in lipopolysaccharide (LPS) synthesis, amongst others, were found in cells grown in the presence of copper. In addition, determination of the relative amounts of LPS present in the cells of each *A. ferrooxidans* strain also supports the idea that these polymers may also have an important role in copper resistance in these biomining bacteria.

## 2. Materials and Methods

### 2.1. Bacterial Strains and Growth Conditions

*A. ferrooxidans* strains ATCC 53993 and ATCC 23270 were grown at 30 °C in liquid 9 K medium containing ferrous sulfate (33.33 g/L) with an initial pH of 1.45 as previously described [23] and in absence or presence of CuSO_4_. Copper concentrations between 40 and 300 mM were used depending on the experiment. In some experiments, concentrations of 100 or 200 mM were used for *A. ferooxidans* ATCC 53993 without prior adaptation since under these two conditions, similar cells numbers to control cells (in absence of copper) were obtained at their respective late exponential growth phases. At 300 mM copper strain ATCC 53993 required previous adaptation. On the other hand, to compare the effect of the metal at the same copper concentration in both strains, it was necessary to adapt strain ATCC 23270 to grow at 100 mM copper. For LPS determinations, strain ATCC 23270 was adapted to grow at 100 mM and strain ATCC 53993 to 200 mM copper. These adaptations were done starting with cells grown at 50 mM copper by increasing 5 mM copper in each successive culture until the desired concentrations were reached. After cells attained late exponential growth phases they were collected and triplicate separate cultures were employed for all experiments. Bacterial growth was determined by measuring the increase in cell numbers by using an Olympus BX50 optical microscope (Olympus, Tokyo, Japan) and a Petroff–Hausser counting chamber (Horsham, PA, USA).

### 2.2. Preparation of Total Protein Extracts for iTRAQ Analysis

*A. ferrooxidans* ATCC 53993 was grown with ferrous iron as oxidizable substrate until late exponential phase in absence or presence of 100 or 200 mM CuSO_4_. Cells were harvested by centrifugation (4000× *g* for 15 min) and washed three times by centrifugation at 4 °C with dilute sulfuric acid (pH 1.5). This was followed by three washes with 50 mM sodium citrate, pH 7.0 by centrifugation at 4 °C to remove any minor ferrous iron remaining and at the same time, to neutralize the pH before cell rupture by sonic oscillation. Cells were then resuspended in sonication buffer (50 mM Tris-HCl pH 8.0, 1 mM ethylenediaminetetra-acetic acid (EDTA) containing phenylmethylsulfonyl fluoride (PMSF) as protease inhibitor (100 μg/mL) and were disrupted by sonic oscillation during 25 min on ice by using successive 5 s pulses and pauses. Finally, the lysate was centrifuged at 10,000× *g* for 10 min to remove unbroken cells and cell debris and the total protein amount in the cell-free extract was determined [21].

### 2.3. Protein Digestion and Tagging with iTRAQ-8-Plex^®^ Reagent

Total protein concentration was determined using microBCA protein assay kit (Pierce, Appleton, WI, USA). For digestion, 50 μg of protein from each condition was precipitated by the methanol/chloroform method. Protein pellets were resuspended and denatured in 20 µL of 7 M urea, 2 M thiourea, 100 mM TEAB (triethylammonium bicarbonate), reduced with 2 µL of 50 mM Tris 2-carboxyethyl phosphine (TCEP) (AB SCIEX, Foster City, CA, USA), pH 8.0, at 37 °C for 60 min and followed by 2 µL of 200 mM cysteine-blocking reagent methyl methanethiosulfonate (MMTS) (Pierce) for 10 min at room temperature. Samples were diluted up to 120 µL with 50 mM TEAB to reduce the concentration of urea. Two µg of sequence grade-modified trypsin (Sigma-Aldrich, St. Louis, MO, USA) was added to each sample (ratio 1:25 enzyme:sample, which were then incubated at 37 °C overnight on a shaker. After digestion, samples were dried in a SpeedVac (Thermo Scientific, Waltham, MA, USA).

Each sample was reconstituted with 180 μL of 70% ethanol/50 mM TEAB, the different versions of the iTRAQ reagent 8-plex (AB SCIEX) were added in additional 20 µL and the mixture was incubated for 2 h at room temperature, according to the following labeling scheme: iTRAQ 113/117 reagent: control 1 and control 2 *A. ferrooxidans*; iTRAQ 115/119 reagent: *A. ferrooxidans* grown in 100 mM CuSO_4_, 1 and grown in 100 mM CuSO_4_, 2; iTRAQ 116/121 reagent: *A. ferrooxidans* grown in 200 mM CuSO_4_, 1 and grown in 200 mM CuSO_4_, 2. Two biological replicas were used in each case. After labeling, samples were combined and the reaction was stopped by evaporation in the SpeedVac.

### 2.4. Liquid Chromatography and Mass Spectrometry Analysis

A 2-µg aliquot of the combined sample was subjected to 2D-nano Liquid Chromatography-Electrospray Ionization Tandem Mass Spectrometry LC ESI-MSMS analysis using a nano liquid chromatography system nanoLC Ultra 1D plus, (Eksigent Technologies, AB SCIEX) coupled to a Quadrupole time of flight (QTOF) type, high speed Triple TOF 5600 mass spectrometer (AB SCIEX) equipped with a nanospray source. Injection volume was 5 µL and three independent technical replicas were analyzed. The analytical column used was a silica-based reversed phase Acquity UPLC Peptide BEH C18 column 75 µm × 15 cm, 1.7 µm particle size and 130 Å pore size (Waters, Dublin, Ireland). The trap column was a C18 Acclaim PepMap (Eksigent Technologies, AB SCIEX), 100 µm × 2 cm, 5 µm particle diameter, 100 Å pore size, switched on-line with the analytical column. The loading pump delivered a solution of 0.1% formic acid in water at 2 µL/min. The nano-pump provided a flow-rate of 300 nL/min and was operated under gradient elution conditions, using 0.1% formic acid in water as mobile phase A, and 0.1% formic acid in acetonitrile as mobile phase B. Gradient elution was performed according to the following scheme: Isocratic conditions of 96% A: 4% B for 5 min, a linear increase to 40% B in 205 min, then a linear increase to 90% B for 15 additional minutes, isocratic conditions of 90% B for 10 min and return to initial conditions in 2 min. Total gradient length was 250 min.

Data acquisition was performed with a TripleTOF 5600 System (AB SCIEX). Ionization occurred under the following conditions: Ionspray voltage floating (ISVF) 2800 V, curtain gas (CUR) 20, interface heater temperature (IHT) 150, ion source gas 1 (GS1) 20, declustering potential (DP) 85 V. All data was acquired using information-dependent acquisition (IDA) mode with Analyst TF 1.5 software (AB SCIEX). For IDA parameters, 0.25 s MS survey scan in the mass range of 350–1250 Da were followed by 25 MS/MS scans of 150 ms in the mass range of 100–1500 (total cycle time: 4 s). Switching criteria were set to ions greater than mass to charge ratio (*m*/*z*) 350 and smaller than *m*/*z* 1250 with charge state of 2–5 and an abundance threshold of more than 90 counts (cps). Former target ions were excluded for 20 s. IDA rolling collision energy (CE) parameters script was used for automatically controlling the CE.

### 2.5. Data Analysis and Statistics

MS/MS spectra were exported to Mascot generic format (mgf) using Peak View v1.2.0.3 and searched using OMSSA 2.1.9, X!TANDEM 2013.02.01.1, Myrimatch 2.2.140 and MS-GF+ (Beta v10072) [24] against a composite target/decoy database built from the 2748 *A. ferrooxidans* sequences at UniprotKB (June 2014). Search engines were configured to match potential peptide candidates with mass error tolerance of 25 ppm and fragment ion tolerance of 0.02 Da, allowing for up to two missed tryptic cleavage sites and a maximum isotope error (13C) of 1, considering fixed MMTS modification of cysteine and variable oxidation of methionine, pyroglutamic acid from glutamine or glutamic acid at the peptide N-terminus, and modification of lysine and peptide N-terminus with iTRAQ 8-plex reagents. Score distribution models were used to compute peptide-spectrum match *p*-values [24], and spectra recovered by a false discovery rate (FDR) ≤ 0.01 (peptide-level) filter were selected for quantitative analysis. Approximately 5% of the signals with the lowest quality were removed prior to further analysis. Differential regulation was measured using linear models [25], and statistical significance was measured using *q*-values (FDR). All analyses were conducted using software from Proteobotics (Madrid, Spain) [24].

### 2.6. Extraction of Total RNA from Acidithiobacillus ferrooxidans and Complementary DNA Synthesis

To determine the effect of copper on the expression of some genes of interest, *A. ferrooxidans* ATCC 23270 and ATCC 53993 cells were grown in absence or presence of CuSO_4_ until cells reached late exponential phase of growth. At this time, total RNA was extracted from each culture condition by lysing the cells as previously reported [26], except that TRIzol (Invitrogen, Carlsbad, CA, USA) was used for the extraction [27,28]. Between three to five biological replicas were used for each experimental condition. Any remaining DNA was eliminated from RNA preparations by addition of 4 U of TURBO DNA-free DNase (Ambion, Thermo Scientific) following manufacturer’s instructions. For complementary DNA (cDNA) synthesis, 0.8 μg of total RNA was reverse transcribed for 1 h at 42 °C using ImProm-II (Promega, Madison, WI, USA) reverse transcription system, 0.5 μg of random hexamers (Promega) and 3 mM MgCl_2_ [28].

### 2.7. Primer Design, Real-Time PCR and Cloning of A. ferrooxidans Genes

Primers for quantitative real time PCR (qRT-PCR) were designed using the Primer3 software [29]. After separating PCR products by electrophoresis in a 1% agarose gel (0.5× Tris–acetate–EDTA pH 8.0 buffer), no cross-amplification or non-specific bands were detected. Copper-resistance related gene expression was analyzed by qRT-PCR with either the Corbett Rotor Gene 6000 system as described previously [21] or with the 96-well PikoReal Real-Time PCR System and Thermo Scientific PikoReal Software 2.2. Efficiency of each primer pair was calculated from the average slope of a linear regression curve, which resulted from qPCRs using a 10-fold dilution series (10 pg–10 ng) of *A. ferrooxidans* DNA as template. Efficiencies between 90 and 110% were used. Quantification cycle (Cq) values were automatically determined by Real-Time Rotor-gene 6000 PCR software (Corbett Life Sciences, Thermo Scientific/Qiagen, Hilden, Germany) or by Thermo Scientific PikoReal Software 2.2.

For transcriptional analysis of the different genes studied, a relative quantification method was used which is based in the ratio between the transcripts of a study sample (in presence of copper) versus a control sample (no copper) [30]. 16S rRNA*_Af_* was selected as a reference gene since its expression was found to be the most stable under our experimental conditions. To carry out the real-time PCR, 0.5 µL of 1:20 diluted cDNA or 0.5 µL of 1:200 diluted 16S rRNA*_Af_*, 0.2 µL of each primer (10 µM) and 5.0 µL of master mix Rotor-Gene SYBR Green PCR (Qiagen) in a final volume of 10 µL, completed with RNA-free water were used. The program used was 10 min at 95 °C followed by 40 cycles of 5 s at 95 °C and 20 s at 60 °C.

### 2.8. Cloning A. ferrooxidans Genes in an Expression Vector

The functionality of different putative copper resistance genes from *A. ferrooxidans* was tested by using heterologous expression in *Escherichia coli*. A copper-sensitive *E. coli* K-12 (*ΔcopA/ΔcusCFBA/ΔcueO*) mutant was transformed with vector pTrc-His2A (Invitrogen) containing the genes of interest under the control of a promoter induced by IPTG, and minimal inhibitory concentration (MIC) values of these transformants were determined as described before [14,28].

### 2.9. Lipopolysaccharide Extraction

*A. ferrooxidans* cells grown in absence or presence of CuSO_4_ were harvested by centrifugation (10,000× *g* for 5 min, at 4 °C). Cell pellets were washed twice with sulfuric acid solution (pH 1.5) and twice with 10 mM sodium citrate (pH 7) by resuspension followed by centrifugation (9200× *g* for 1 min). Cells were then resuspended in sulfuric acid solution. To normalize the number of cells, optical density of cell suspensions was measured at 600 nm, adjusting them to an optical density of 2 (OD_600nm_ = 2) in 1 mL of sulfuric acid solution. Cell suspensions were then centrifuged at 10,000× *g* for 5 min. A partially modified Hitchcock & Brown method for LPS extraction was used [31]. The cell pellet was resuspended in 90 µL of lysis buffer solution (2% Sodium dodecyl sulfate (SDS); 4% 2-ME; 0.5 M Tris-HCl, pH 9.0). The suspension was heated for 30 min at 100 °C. Lysed cells were then digested with 100 µg/mL of DNase I (Ambion) for 90 min at 37 °C. Samples were thereafter treated with 1 mg/mL of Proteinase K (Sigma-Aldrich) for 90 min at 60 °C. Finally, samples were dialyzed for 30 min against nano-pure water using a nitrocellulose membrane, 0.025 µm pore size. Dialyzed samples were finally stored at 4 °C for further analysis.

### 2.10. Lipopolysaccharide Quantification

Extracted LPS was quantified by purpald assay [32]. Unsubstituted terminal vicinal glycol (UTVG) groups of the sugar residues such as Kdo and heptose in LPS can be subjected to periodate oxidation, yielding quantitative formaldehyde measurable by the purpald reagent. This assay provides the molarity of the UTVG present in LPS. LPS molarity can be found by dividing the molarity of the UTVG by the theoretical number of UTVG per LPS molecule. The numbers of UTVG present in LPS of *A. ferrooxidans* is unknown. Therefore, LPS concentration was expressed in relation to the molarity of UTVG present in each sample. The experimental procedure was carried out as previously described [33].

## 3. Results and Discussion

### 3.1. Proteomic Analysis of the Copper Response of A. ferrooxidans ATCC 53993

Proteins of cells grown in ferrous iron and in presence of 100 or 200 mM CuSO_4_ were analyzed by quantitative iTRAQ proteomics. In cells subjected to 100 mM of copper 1656 proteins were identified, of which 28 changed their levels compared to control cells grown in absence of copper. Of these proteins, 11 had higher levels and 17 showed lower amounts (Table S1). On the other hand, in cells grown in 200 mM copper 1567 proteins were identified and 59 of these showed changes in their levels compared to the control. Seventeen showed higher levels than the control and 42 lower amounts (Table S1). This corresponds to about 2-fold more proteins changing at 200 mM than at 100 mM copper (Table S1). Most of the proteins changing at 100 mM copper were also seen to vary at 200 mM of the metal (Tables S2 and S3). The functional categories of all proteins changing in *A. ferrooxidans* 53993 are shown in Table S1 and Figure S1 and the data obtained is seen in Tables S2 and S3. Although at 200 mM copper ATCC 53993 cells grew reaching similar numbers to the control, they were apparently more affected than cells subjected to 100 mM copper since a greater number of proteins related to metabolism and protein biosynthesis decreased their levels whereas others related to energy production and copper resistance increased their amounts. Nevertheless, at 200 mM copper cells are still actively expressing the proteins related to the RND efflux systems (Table 1), as seen before at 40 mM copper sulfate [22]. In addition, an interesting group of proteins that also form part of the cell envelope changed their synthesis levels in presence of the metal (Table 1). Most of these proteins may be new possible copper resistance determinants present in *A. ferrooxidans* ATCC 53993.

### 3.2. Overexpression of Resistance-Nodulation-Division Efflux Transporters and Possible Generation of Excess Acidity

**Figure 1:**
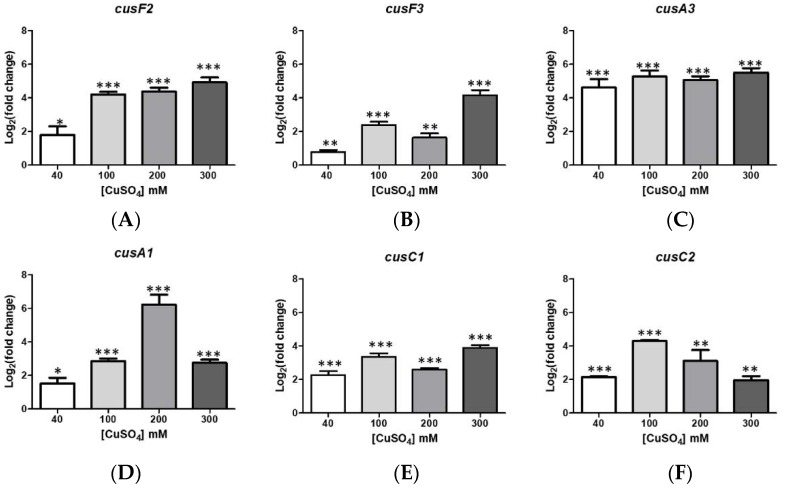
Transcriptional levels of several genes coding for Cus system components in *Acidithiobacillus ferrooxidans* ATCC 53993. The transcriptional levels of genes (**A**) *cusF2*; (**B**) *cusF3*; (**C**) *cusA3*; (**D**) *cusA1*; (**E**) *cusC1* and (**F**) *cusC2* were determined at the indicated copper concentrations as described in Material and Methods section. Error bars indicate the standard deviations based on three different experimental values. Application of *t-*Student test were: *** *p* ≤ 0.001 ** *p* ≤ 0.01 and * *p* ≤ 0.05.

Transcriptional levels of some genes coding for possible components of the RND family of efflux transporters [10] are shown in Figure 1. These correspond to most of the genes coding for Cus system components in both *A. ferrooxidans* ATCC 53993 [20,22] and ATCC 23270 [28]. All these genes showed increased transcriptional levels in cells grown at the indicated copper concentrations. These Cus transporter systems are widely present in bacteria to remove copper from the cell [10]. *A*. *ferrooxidans* lives at an acid external pH (1–3) and its cytoplasmic pH is up to 5 units higher than external pH. This generates an elevated pH gradient across the cytoplasmic membrane that contributes to the proton motive force (PMF) comprising membrane potential (ΔΨ) and transmembrane pH difference (ΔpH) [22,34]. RND type transporters are antiporters taking advantage of the proton gradient to efflux copper with the concomitant protons entrance to the cytoplasm. Due to its economy from the energetic point of view, these systems would be used preferentially by acidophilic microorganisms to remove intracellular copper.

**Figure 2:**
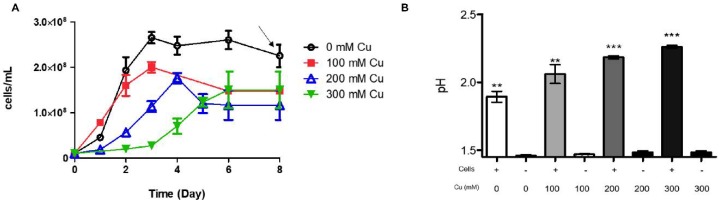
Growth medium pH changes of *A. ferrooxidans* ATCC 53993 grown in absence or presence of copper. (**A**) Cells were grown in ferrous iron medium at the indicated copper concentrations. Once cells reached stationary phase of growth (indicated by the arrow), aliquots of the cultures were taken and centrifuged to remove cells; (**B**) pH values of the media supernatants were determined and compared with pH changes of the medium containing the same copper concentrations but in absence of inoculated cells. Error bars indicate standard deviations based on three different experimental values. Application of *t*-Student test were: *** *p* ≤ 0.001 ** *p* ≤ 0.01 and * *p* ≤ 0.05.

A possible cytoplasmic acidification would be expected if these efflux pumps were excessively used by *A. ferrooxidans* in presence of high metal concentrations. Conversely, as previously pointed out [22], this acidification could be diminished by the energetic metabolism of the bacterium, since oxidation of Fe(II) by molecular oxygen as the final electron acceptor consumes protons [35]. Still, RND systems may introduce an excess of protons from the acid culture medium to the cell during copper detoxification. This idea has not been demonstrated in *A. ferrooxidans*. However, a possible increase in the extracellular pH of the growth medium could be expected during growth in the presence of copper. Figure 2A shows growth curves of *A. ferrooxidans* ATCC 53993 and how cell growth was affected at the indicated copper concentrations. Initially, there was a strong partial inhibition of growth only at 300 mM copper.

Figure 2B clearly shows an increase in external pH of the growth medium (reaching around 0.6 pH units at 300 mM copper). On the contrary, pH values of control media containing different copper concentrations and no cells inoculated showed only very minor pH variations. Clearly, whether this interesting preliminary observation is due to intracellular acidification remains to be demonstrated.

### 3.3. Changes of Several Additional Envelope Components Occur in Presence of High Copper Concentrations

Another protein with increased levels in cells grown in presence of copper was a putative periplasmic glucan biosynthesis protein MdoG coded by *Lferr_1075* (Table 1). This protein is involved in the synthesis of ramifications present in the osmoregulated periplasmic glucans (OPGs) in bacteria. These OPGs are present in all known proteobacteria and are formed by 5–24 d-glucose molecules bound by means of β-glycosidic bonds. The concentration of these glucans has been reported to change with variations in periplasmic osmolarity [36]. Due to their big size, OPGs are trapped in the periplasm, being unable to diffuse to the outside of cells. In *E. coli*, the carbon skeleton is synthesized by proteins coded by genes *opgG* (*mdog* orthologous) and *opgH*. OpgH is a glucosyl transferase that synthesizes the lineal skeleton of glucose units by means of β-1,2 bonds. In *E. coli*, MdoG is a 56 kDa periplasmic protein necessary for the polymerization of sugar molecules, although its function has not being completely established [36]. In *A. ferrooxidans*, putative MdoG (57.4 kDa) has been previously identified as a component of its periplasm [37]. In *E. coli* both *opgG* and *opgH* genes form part of the same operon. By analyzing the genomic context of *A. ferrooxidans* instead, it was found that *mdoG* and *mdoH* are separated by an open reading frame (ORF) coding for a protein of unknown function. On the other hand, protein MdoH did not change its levels in the results obtained here. The system for OPGs synthesis in *E. coli* involves four additional proteins (OpgD, OpgB, OpgC and OpgE) whose equivalent genes are absent in *A. ferrooxidans* genome. Nevertheless, only OpgG and OpgH are strictly necessary for the OPGs synthesis in *E. coli* [38]. OPGs biosynthesis starts with glucose transport to form glucose-6P, which is used to generate (uridine diphosphate glucose) UDP-glucose for production of OPGs via OpgH/OpgG [36]. It is known this molecule is formed by a glucose 1-phosphate uridil transferase that catalyzes the UTP and a proton addition to d-glucose-1-phosphate to generate UDP-d-glucose [39]. It can be suggested that generation of OPGs in *A. ferrooxidans* would involve also a higher UDP-glucose synthesis. Since this process consumes protons, it should alleviate excessive entrance of these cations when RND efflux pumps are heavily used to remove copper.

The CusA proton/Cu antiporter system is overexpressed in *A. ferrooxidans* subjected to copper as already seen in Figure 1, and under those conditions, a higher number of protons would be expected to enter the cytoplasm from the growth medium, as already suggested by the results shown in Figure 2. Thus, a higher synthesis of OPGs would also consume protons, in favor of keeping the normal cytoplasmic pH. Furthermore, it has been shown that an OPGs preparation acts as a blocker and a regulator of an OMPC-like porin channel selective of cations in *E. coli* [40]. On the other hand, cells unable to form OPGs showed an increased synthesis of OmpC [41]. It has also been documented that porins mediate copper entrance in *Mycobacterium tuberculosis* [42]. The existence of a relationship between both copper entrance and porins closing or decreasing their levels of synthesis is possible then, as seen here for OprB in *A. ferrooxidans* ATCC 53993 (Table 1). Examples of this behavior were previously reported for the major *A. ferrooxidans* porin Omp40 (*Afe_2741*) and OmpA (*Afe_2685*) in *A. ferrooxidans* ATCC 23270 [21].

**Figure 3:**
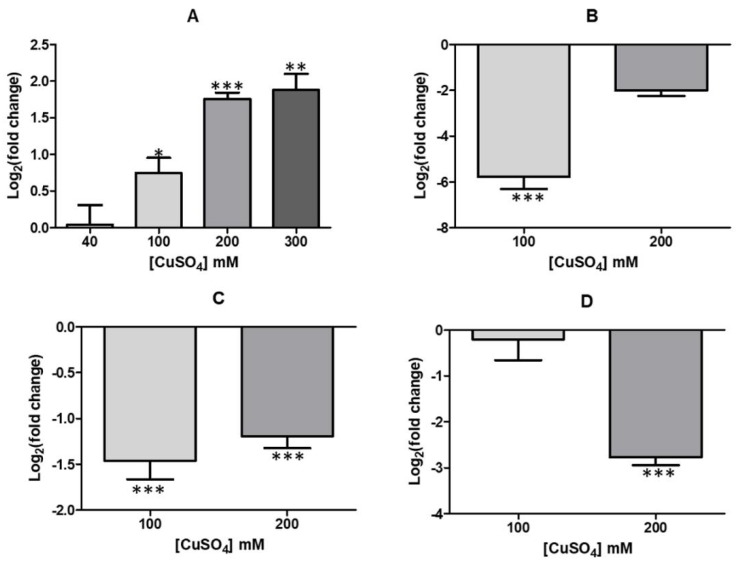
Transcriptional levels of selected envelope genes. (**A**) *mdoG*; (**B**) *oprB*, (**C**) *ompA* and (**D**) *omp40* in *A. ferrooxidans* ATCC 53993 grown in different copper concentrations. Error bars indicate standard deviations based on three different experimental values. Application of *t-*Student test were: *** *p* ≤ 0.001 ** *p* ≤ 0.01 and * *p* ≤ 0.05.

To support proteomic results, transcriptional levels of genes coding for proteins MdoG and porins, were also determined in cells grown at different copper concentrations as shown in Figure 3. The results clearly indicate increasing levels of synthesis of mRNA coding for MdoG and decreasing levels of messenger RNAs for porin genes *omp40*, *oprB* and *ompA*, confirming the proteomic results already discussed. It is, therefore, possible that lower levels of porins, together with higher OPGs amounts, constitute a defense response to extreme copper conditions as seen here in *A. ferrooxidans* ATCC 53993, an idea that should be proven.

**Figure 4:**
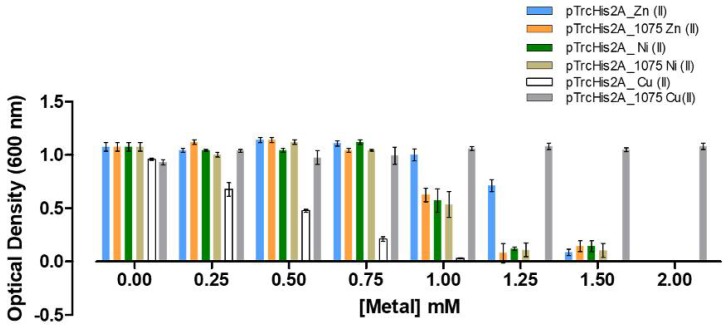
Heterologous functional analysis of the overexpression of *A. ferrooxidans mdoG* (*Lferr_1075*) gene in the Cu-sensitive *Escherichia coli* (K-12 *ΔcopA/ΔcusCFBA/ΔcueO*) grown in zinc (Zn), nickel (Ni) and copper (Cu). pTrcHis2A, empty vector; pTrcHis2A_1075 contains *mdoG* gene. Error bars indicate standard deviations based on three different experimental values.

Currently there are no efficient and easy to reproduce methods to generate knock-outs of genes in *A. ferrooxidans* [43]. Therefore, to ascertain whether the *mdoG* gene confers Cu-resistance to a heterologous host, it was expressed in *E. coli* as described in Materials and Methods. As seen in Figure 4, *A. ferrooxidans* putative *mdoG* gene conferred resistance to Cu when expressed in *E. coli* due to the increase of its MIC value from 1.0 to 3.0 mM copper. This result supports the possibility of MdoG being a copper resistance determinant in this acidophilic microorganism. In addition, the effect of overexpressing *mdoG* in *E. coli* was also tested in cells grown in the presence of Zn or Ni as shown in Figure 4.

**Figure 5:**
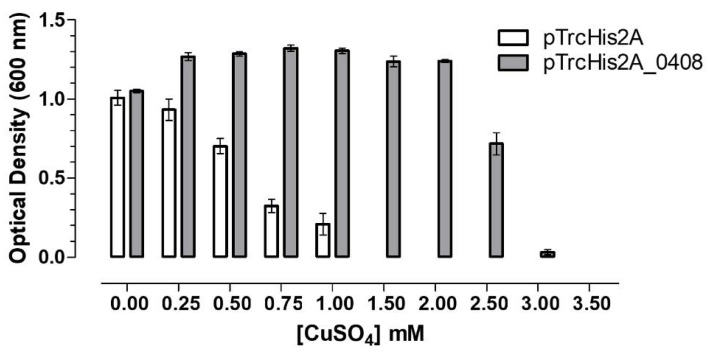
Heterologous functional analysis of overexpression of *A. ferrooxidans* antigen-O polymerase gene *wzy* (*Lferr_0408*) in Cu-sensitive *E. coli* (K-12 *ΔcopA/ΔcusCFBA/ΔcueO*) grown in copper. pTrcHis2A, empty vector; pTrcHis2A_0408 contains gene *wzy*. Error bars indicate standard deviations based on three different experimental values.

Compared with copper, MdoG did not confer tolerance to Ni and Zn. Interestingly, a proteomic analysis of the response of *Rhodobacter sphaeroides* to high cobalt concentrations has been reported [44]. One of the changing proteins in presence of the metal was MdoG. It was previously suggested that cobalt would generate an alteration of permeability of the envelope, periplasm or cell wall as a possible resistance mechanism in this microorganism [44]. Whether the effect of copper is rather specific for MdoG from *A. ferrooxidans* remains to be elucidated. Another interesting protein found to be overexpressed in presence of copper was *Lferr_0408* (Wzy) (Table 1), a protein involved in O-antigen biosynthesis, the most external segment of LPS [45,46]. By expressing gene *wzy* from *A. ferrooxidans* in the Cu-sensitive *E. coli* (K-12 Δ*copA/ΔcusCFBA/ΔcueO*) strain already used for *mdoG* gene, the results seen in Figure 5 were obtained. Once again, it is clear that expressing *wzy* gene in the heterologous host confers it a higher copper resistance.

**Figure 6:**
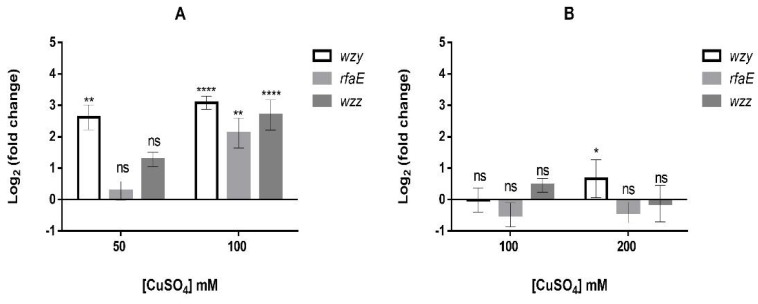
Transcriptional levels of genes *wzy, wzz* and *rfaE* related to lipopolysaccharides (LPS) synthesis in *A. ferrooxidans* exposed to copper. (**A**) Strain ATCC 23270; (**B**) Strain ATCC 53993. Values were obtained from three biological replicates. Error bars indicate standard deviations based on three different experimental values. Application of *t*-Student test were: **** *p* ≤ 0.0001 ** *p* ≤ 0.01 and * *p* ≤ 0.05.

To support this result, the analysis of transcriptional expression of this and other genes involved in LPS generation was carried out. Figure 6 shows the levels of transcriptional expression of *wzy, wzz* and *rfaE* genes, all involved with LPS synthesis, in both *A. ferrooxidans* strains grown in the absence or presence of copper.

**Figure 7:**
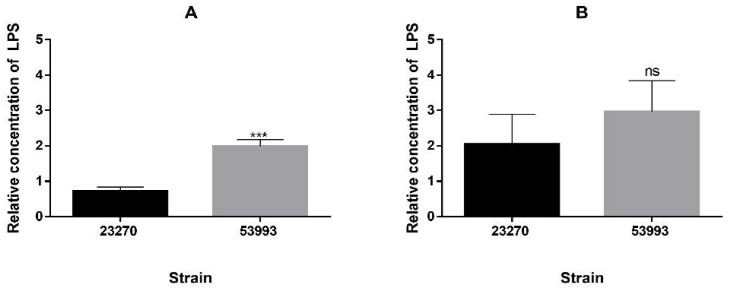
Relative LPS concentration of *A. ferrooxidans* ATCC 23270 and ATCC 53993 grown at different concentrations of CuSO_4_. (**A**) Cells grown in absence of copper; (**B**) Cells grown in 100 mM CuSO_4_. Values were obtained from three biological replicates. Error bars represent standard deviations for each condition. A *t*-Student statistic analysis was performed, where: *** indicates *p* ≤ 0.001 and ns indicates *p* > 0.05.

Strain ATCC 23270 clearly showed an increased level in expression of tested genes in presence of the metal (Figure 6A). On the contrary, the same genes did not show significant changes in their expression when strain ATCC 53993 was grown in presence of copper (Figure 6B). These results strongly suggest strain ATCC 23270 could synthesize higher amounts of LPS in presence of copper compared to ATCC 53993. However, when the amounts of LPS were determined in both strains in absence of the metal, ATCC 53993 showed about 3-fold higher amounts of LPS compared with ATCC 23270 (Figure 7A). This result indicates that normally, strain ATCC 53993 in addition of having extra copper resistance determinants in its genomic island, contains higher LPS levels compared with strain ATCC 23270. This could explain in part the higher copper tolerance of the former strain.

**Figure 8:**
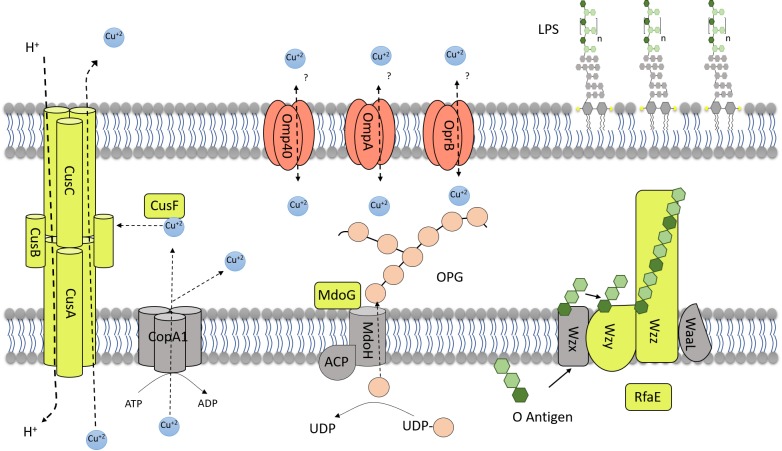
Summary working model of some proteins in *A. ferrooxidans* adapted to grow in presence of copper mentioned in this study. Yellow, proteins that increase their synthesis or transcript levels in presence of copper. Pink, proteins down-regulated in cells subjected to copper. OPG stands for osmoregulated periplasmic glucans. The locations and order in which proteins are illustrated are arbitrary.

Nonetheless, in presence of 100 mM copper both strains showed similar LPS levels (Figure 7B), suggesting that strain 23270 increases its LPS levels in presence of the metal, in agreement with results of the transcriptional expression of its genes in the presence of copper seen in Figure 6. Previously, *A. ferrooxidans* ATCC 53993 subjected to 40 mM Cu showed an increased level of protein RfaE possibly involved in LPS synthesis [22]. Apparently, LPS could bind metals in the cell surface depending on the composition of the polymers [47]. A summary of the main results obtained is shown in the working model of Figure 8.

Remarkably, it has been reported that *A. ferrooxidans* adapted to high copper and zinc ions concentrations showed changes in the surface chemical properties of this bacterium. Under these conditions, their surface negative charge was decreased due to changes in the structure of its surface layers [48].

## 4. Conclusions

The results presented here clearly indicate that several envelope components from *A. ferrooxidans* such as RND efflux pumps, LPS, porins, and periplasmic protein MdoG may be of great relevance for both, copper resistance and/or tolerance in their environment. Similar roles for these components in other members of the biomining bacterial consortia are also possible and their study may be of importance for industrial bioleaching operations.

## Figures and Tables

**Table 1:** Levels of some selected known and new possible copper resistance determinants in *A. ferrooxidans* ATCC 53993 grown in the presence of 200 mM CuSO_4_.

Function/Similarity	ORF	Name	q Value (FDR)	Coverage (%)	Peptide Number	Log_2_ Fold Change (Cu 200/0 mM)
Outer membrane efflux protein	*Lferr_1619*	CusC1	0.001	45	9	1.258
Efflux transporter, RND family, MFP subunit	*Lferr_1618*	CusB1	0.001	63.3	15	0.859
Uncharacterized protein	*Lferr_2057*	CusF2	0.001	60	3	1.92
Uncharacterized protein	*Lferr_0174*	CusF3	0.001	60	3	1.63
Heavy metal efflux pump, CzcA family	*Lferr_0172*	CusA3	0.001	39.1	10	1.019
Outer membrane efflux protein	*Lferr_2062*	CusC2	0	45	12	1.084
Heavy metal efflux pump, CzcA	*Lferr_1617*	CusA1	0.002	35	9	0.855
Efflux transporter, RND family, MFP subunit	*Lferr_2061*	CusB2	0.003	68.3	6	0.968
Heavy metal efflux pump, CzcA family	*Lferr_2060*	CusA2	0.003	38.3	9	0.846
Periplasmic glucan biosynthesis protein MdoG	*Lferr_1075*	MdoG	0.009	48.1	15	0.415
Carbohydrate-selective porin OprB	*Lferr_1898*	OprB	0.005	36.46	10	−0.635
O-antigen polymerase	*Lferr_0408*	Wzy	0.026	3.19	1	1.795

## Supplementary Materials

The following are available online at http://www.mdpi.com/2073-4425/9/7/347/s1. Figure S1: Functional categories of *A. ferrooxidans* proteins changing their synthesis levels in cells grown in presence of 100 and 200 mM CuSO_4_. Table S1: Functional categories and numbers of *A. ferrooxidans* ATCC 53993 proteins changing their synthesis levels in cells grown in presence of 100 and 200 mM CuSO_4_. Table S2: Proteins with increased levels in *A. ferrooxidans* ATCC 53993 grown in presence of 100 and 200 mM CuSO_4_. Table S3: Proteins with lower levels of synthesis in *A. ferrooxidans* ATCC 53993 grown in presence of 100 and 200 mM CuSO_4_.
