# Supplementary material for: Possible Role of Envelope Components in the Extreme Copper Resistance of the Biomining Acidithiobacillus ferrooxidans
Source: Genes (Basel). 2018 Jul 10;9(7):347. doi: 10.3390/genes9070347 (PMC6070983; doi:10.3390/genes9070347)

Supplementary Information

Possible Role of envelope components in the extreme copper resistance of the biomining *Acidithiobacillus ferrooxidans*

Nia Oetiker[1], Rodrigo Norambuena[1], Cristóbal Martínez-Bussenius[1], Claudio A. Navarro[1], Fernando Amaya[2], Sergio A. Álvarez[2], Alberto Paradela[3] and Carlos A. Jerez[1*]

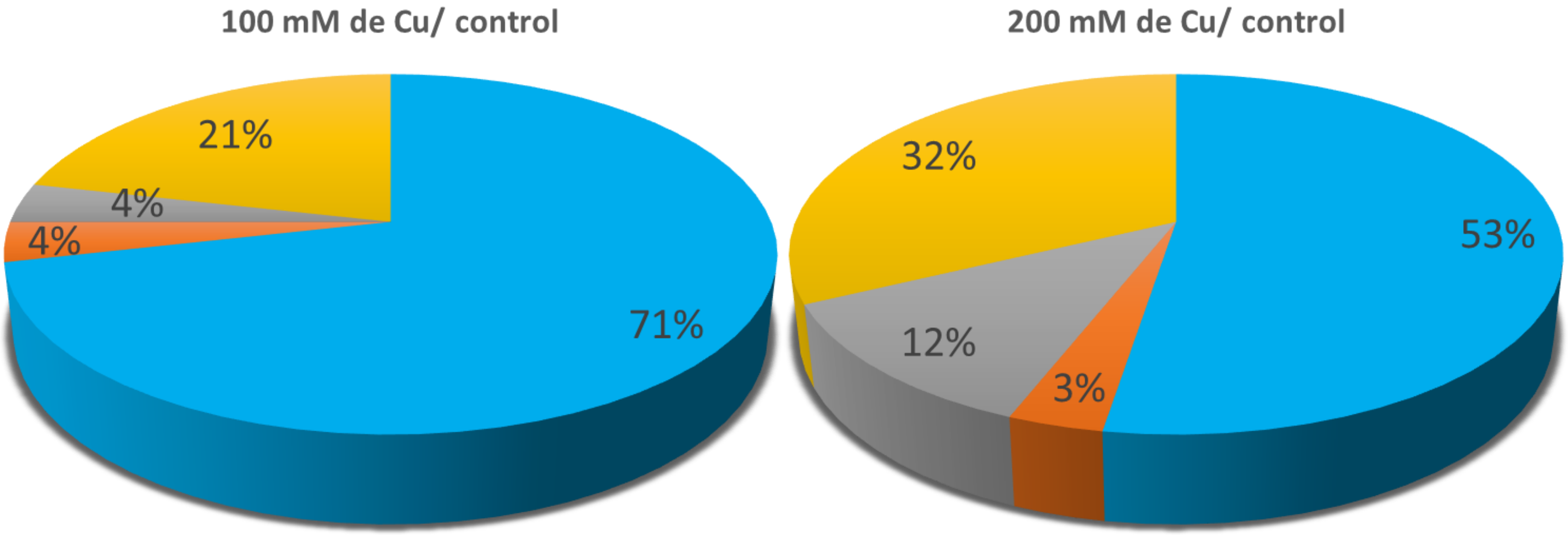

Figure S1. Functional categories of *A. ferrooxidans* proteins changing their synthesis levels in cells grown in the presence of 100 and 200 mM $CuSO_4$. Blue: Metabolism; Orange: Cellular Processes and Signaling; Gray: Information Storage and Processing; Yellow: Poorly Characterized

Table S1. Functional categories and numbers of *A. ferrooxidans* ATCC 53993 proteins changing their synthesis levels in cells grown in the presence of 100 and 200 mM $CuSO_4$.

	Number of proteins changed			
Functional category	100 mM de Cu/ Control		200 mM de Cu /Control	
	Protein (-)	Protein (+)	Protein (-)	Protein (+)
**METABOLISM**				
Inorganic Ion Transport and Metabolism	3	9	5	10
Carbohydrate Metabolism and Transport	5	0	5	1
Energy Production and Conversion	1	0	4	4
Secondary Metabolites biosynthesis, Transport, and Catabolism	1	0	2	0
Lipid transport and metabolism	1	0	0	0
**INFORMATION STORAGE AND PROCESSING**				
Translation, Ribosomal structure and Biogenesis	0	1	7	0
**CELLULAR PROCESSES AND SIGNALING**				
Post-translational Modification, Protein turnover, and Chaperones	1	0	1	0
Cell wall/Membrane/Envelope biogenesis	0	0	1	0
**POORLY CHARACTERIZED**				
General function prediction only	0	0	1	0
Function Unknown	5	1	16	2
Total	17	11	42	17

(+) increased level. (−) decreased level.

Table S2. Proteins with increased levels in *A. ferrooxidans* ATCC 53993 grown in the presence of 100 and 200 mM $CuSO_4$

Accession number	ORF	Function/similarity	qvalue (FDR)	Coverture (%)	Peptides number	Log2 Fold change (Cu 100/0 mM)	Log2 Fold change (Cu 200/0 mM)
**METABOLISM**							
**Inorganic ion transport and metabolism**							
B5EJG7	*Lferr_1619*	Outer membrane efflux protein (CusC1)	0.001	45	9	1.018	1.258
B5EJG6	*Lferr_1618*	Efflux transporter, RND family, MFP subunit (CusB1)	0.001	63.3	15	0.927	0.859
B5EM36	*Lferr_2057*	Periplasmatic Copper chaperone (CusF2)	0.001	60	3	1.084	1.92
B5EK73	*Lferr_0174*	Periplasmatic Copper chaperonea (CusF3)	0.001	60	3	0.68	1.63
B5EK71	*Lferr_0172*	Heavy metal efflux pump, CzcA family (CusA3)	0.001	39.09	10	1.06	1.019
B5EM41	*Lferr_2062*	Outer membrane efflux protein (CusC2)	0	44.5	12	0.823	1.084
B5EJG5	*Lferr_1617*	Heavy metal efflux pump, CzcA family (CusA1)	0.002	35	9	1.022	0.855
B5EM40	*Lferr_2061*	Efflux transporter, RND family, MFP subunit (CusB2)	0.003	68.3	6	0.801	0.968
B5EM39	*Lferr_2060*	Heavy metal efflux pump, CzcA family (CusA2)	0.003	38.3	9	0.927	0.846
B5EQ72	*Lferr_1075*	Periplasmic glucan biosynthesis protein (MdoG)	0.009	48.1	15	0	0.415
**Carbohydrate metabolism and transport**							
B5EL00	*Lferr_1865*	Carboxymethylenebutenolidase	0.011	88.43	16	0	0.347
**Energy production and conversion**							
B5EJH8	*Lferr_1630*	Cytochrome c class I	0.026	9.64	1	0	1.843
B5EQK0	*Lferr_2705*	Cytochrome c class I (CycA1)	0.013	27.31	5	0	0.515
B5EMP7	*Lferr_2183*	FAD-dependent pyridine nucleotide-disulphide oxidoreductase	0.007	65.53	20	0	0.369
B5ES29	*Lferr_1469*	FAD-dependent pyridine nucleotide-disulphide oxidoreductase	0.015	74.65	21	0	0.341

**INFORMATION STORAGE AND PROCESSING**							
**Translation, ribosomal structure and biogenesis**							
B5ENR6	*Lferr_2358*	Uncharacterized protein	0.014	17.02	1	1.593	0
**POORLY CHARACTERIZED**							
Function unknown							
B5ELE6	*Lferr_0408*	O-antigen polymerase (Wzy)	0.026	3.19	1	0	1.795
B5EM38	*Lferr_2059*	Uncharacterized protein	0.005	38.86	5	0	0.936
B5ELK0	*Lferr_1967*	Uncharacterized protein	0.025	4.17	1	1.081	0

Color represents change in log_2 fold change value: green, increase; yellow, no change; red, decrease.

Table S3. Proteins with lower levels of synthesis in *A. ferrooxidans* ATCC 53993 grown in the presence of 100 and 200 mM $CuSO_4$.

Accession number	ORF	Function/similarity	qvalue (FDR)	Coverture (%)	Peptides number	Log2 Fold change (Cu 100/0 mM)	Log2 Fold change (Cu 200/0 mM)
**METABOLISM**							
**Inorganic ion transport and metabolism**							
B5EJZ3	*Lferr_1702*	TonB-dependent receptor	0.039	43.46	18	0	-0.318
B5EQV4	*Lferr_1216*	Extracellular solute-binding protein family	0.049	69.38	15	-0.463	-0.343
B5ERT7	*Lferr_1375*	von Willebrand factor type A	0.003	64.11	35	0	-0.37
B5EPV1	*Lferr_2552*	TonB-dependent receptor	0.011	26.94	16	0	-0.407
B5EJF9	*Lferr_1611*	Phosphate-binding protein PstS	0.001	58.29	13	-0.934	-0.488
B5EK98	*Lferr_0199*	Periplasmatic Copper chaperone (CusF4)	0.007	51.61	6	-0.673	0
**Carbohydrate metabolism and transport**							
B5ERT6	*Lferr_1374*	Ketose-bisphosphate aldolase class-II	0.007	76.84	22	0	-0.374
B5ERV1	*Lferr_1389*	Ribulose-bisphosphate carboxylase	0	72.94	31	-0.501	-0.574
B5ERV0	*Lferr_1388*	Ribulose-bisphosphate carboxylase	0.001	90	9	-0.501	-0.53
B5ERS6	*Lferr_1364*	Transketolase central region	0.001	65.36	31	-0.699	-1.163
B5ERU9	*Lferr_1387*	Carboxysome structural protein CsoS2	0.002	68.67	47	-0.289	-1.185
B5EL78	*Lferr_0340*	Toluene tolerance family protein	0.024	67.45	9	-0.439	0
**Energy production and conversion**							
B5EQK2	*Lferr_2707*	Ubiquinol-cytochrome c reductase iron-sulfur subunit (petA1)	0.003	45.63	9	0	-0.478
B5ERS3	*Lferr_1361*	FAD linked oxidase domain protein	0.028	23.51	4	0	-0.588

B5ERS1	*Lferr_1359*	NADH/Ubiquinone/plastoquinone (Complex I)	0.003	17.58	7	0	-0.956
B5ERS4	*Lferr_1362*	D-lactate dehydrogenase (Cytochrome)	0.01	8.2	2	0	-1.157
B5EQY4	*Lferr_2743*	Rusticyanin	0.002	74.87	14	-1.139	0
**Secondary metabolites biosynthesis, transport, and catabolism**							
B5ERU3	*Lferr_1381*	Microcompartments protein	0.004	95.54	4	0	-0.69
B5ERU5	*Lferr_1383*	Microcompartments protein	0.022	94.9	1	0	-0.851
B5EKF9	*Lferr_1773*	Hopanoid biosynthesis associated membrane protein HpnM	0.007	55.71	11	-0.485	0
**Lipid transport and metabolism**							
B5EKF4	*Lferr_1768*	Uncharacterized protein	0.011	37.16	5	-0.633	0
**INFORMATION STORAGE AND PROCESSING**							
**Translation, ribosomal structure and biogenesis**							
B5EMD3	*Lferr_0558*	30S ribosomal protein S15	0.004	48.31	2	0	-0.784
B5ELY3	*Lferr_0501*	30S ribosomal protein S19	0.001	70.33	7	0	-0.867
B5EKY1	*Lferr_1846*	30S ribosomal protein S20	0.003	25.27	3	0	-0.932
B5ELZ0	*Lferr_0508*	50S ribosomal protein L24	0.005	19.81	3	0	-0.982
B5ELZ7	*Lferr_0515*	50S ribosomal protein L30	0.002	45	3	0	-1.056
B5ENA6	*Lferr_2294*	50S ribosomal protein L33	0.002	47.06	2	0	-1.513
B5ELU3	*Lferr_0461*	50S ribosomal protein L27	0.044	58.14	5	0	-0.416
**CELLULAR PROCESSES AND SIGNALING**							
**Post-translational modification, protein turnover, and chaperones**							
B5EN20	*Lferr_0699*	10 kDa chaperonin groS	0.007	90.63	11	-0.445	-0.404
**Cell wall/membrane/envelope biogenesis**							
B5EL33	*Lferr_1898*	Carbohydrate-selective porin (OprB)	0.005	36.46	10	0	-0.635
**POORLY CHARACTERIZED**							

			**General function prediction only**					
B5ERT8	*Lferr_1376*	CbbQ/NirQ/NorQ domain protein	0.018	83.15	13	0	-0.348
			**Function unknown**					
B5ERD1	*Lferr_1295*	Uncharacterized protein	0.016	51.28	7	0	-0.458
B5ERS9	*Lferr_1367*	Uncharacterized protein	0.012	28.08	8	0	-0.519
B5EMQ4	*Lferr_2190*	Uncharacterized protein	0.007	56.44	6	0	-0.531
B5EP93	*Lferr_2439*	Uncharacterized protein	0.003	51.37	6	0	-0.569
B5EJI6	*Lferr_0030*	Pyrrolo-quinoline quinone	0.003	43.29	11	-0.468	-0.62
B5ERS0	*Lferr_1358*	UPF0753 protein	0	70.18	51	-0.401	-0.64
B5EQX9	*Lferr_2738*	Uncharacterized protein	0.003	63.43	9	0	-0.649
B5ERS5	*Lferr_1363*	Uncharacterized protein	0.003	64.94	17	0	-0.733
B5ERU8	*Lferr_1386*	Carboxysome shell carbonic anhydrase	0.001	52.72	21	0	-0.772
B5EL34	*Lferr_1899*	Pyrrolo-quinoline quinone	0.001	47.59	15	-0.373	-0.778
B5EM86	*Lferr_2116*	Uncharacterized protein	0.003	28	4	0	-0.909
B5EJU1	*Lferr_1650*	Uncharacterized protein	0.003	27.2	4	0	-0.975
B5EJ87	*Lferr_1539*	Uncharacterized protein	0.011	12.7	1	-2.336	-1.109
B5ELR1	*Lferr_0429*	Uncharacterized protein	0.03	36.89	9	0	-1.124
B5ERT9	*Lferr_1377*	Uncharacterized protein	0.016	10.71	1	0	-1.149
B5EJC5	*Lferr_1577*	Ribonuclease BN	0.046	2.71	1	0	-1.11
B5EN88	*Lferr_2275*	Uncharacterized protein	0.008	54.17	4	-0.818	0

Color represents the change in log_2 fold value: green, increased level; yellow, no change; red, decreased level.

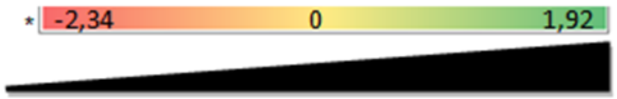

Supplement: Supplementary file 1 [file genes-09-00347-s001.zip › genes-316257-SI.pdf]
